# Blood Pressure Status Modulates the Therapeutic Response to Sodium‐Glucose Cotransporter 2 Inhibitors in Diabetic Macular Edema: A Post Hoc Subgroup Analysis of the COMET Trial

**DOI:** 10.1111/1753-0407.70184

**Published:** 2025-12-18

**Authors:** Ryoichi Ishibashi, Masaya Koshizaka, Yoko Takatsuna, Tomoaki Tatsumi, Takayuki Baba, Shuichi Yamamoto, Koutaro Yokote

**Affiliations:** ^1^ Department of Medicine, Division of Diabetes, Endocrinology and Metabolism Kimitsu Chuo Hospital Chiba Japan; ^2^ Department of Endocrinology, Haematology and Gerontology Chiba University Graduate School of Medicine Chiba Japan; ^3^ Department of Nutrition and Metabolic Medicine, Center for Preventive Medical Sciences Chiba University Chiba Japan; ^4^ Department of Ophthalmology Chiba Rosai Hospital Chiba Japan; ^5^ Department of Ophthalmology and Vision Science Chiba University Graduate School of Medicine Chiba Japan

**Keywords:** anti‐vascular endothelial growth factor injection, diabetic macular edema, hypertension, sodium‐glucose cotransporter 2 inhibitor

## Abstract

**Aims:**

To evaluate the feasibility of sodium‐glucose cotransporter 2 inhibitors (SGLT2i) as a systemic adjunct for patients with diabetic macular edema (DME) and hypertension.

**Materials and Methods:**

This study encompassed a post hoc analysis of the COMET Trial data, focusing on patients with DME and hypertension, defined by office systolic blood pressure (OSBP) ≥ 140 mmHg or a documented history of hypertension. Participants were randomized to receive either SGLT2i (luseogliflozin) or sulfonylurea (SU, glimepiride). The primary outcome was the treatment burden, quantified by the total number of intravitreal ranibizumab injections (IVRs) over 48 weeks.

**Results:**

Within the OSBP ≥ 140 mmHg subgroup, 14 patients received SGLT2i and 15 received SU. The total number of IVRs was 4.1 ± 3.1 in the SGLT2i group and 6.8 ± 3.1 in the SU group (Cohen's *d* = 0.87; power = 0.82). The adjusted analysis of covariance further confirmed significantly fewer IVRs in the SGLT2i group (3.3 ± 1.1 vs. 6.2 ± 1.0, *p* = 0.025). OSBP was significantly reduced in the SGLT2i group at Week 12, but there was no significant difference at Week 48. Office diastolic blood pressure remained consistently lower in the SGLT2i group. No significant differences in IVR frequency were observed in other subgroups.

**Conclusions:**

SGLT2i may help reduce the treatment burden of IVRs in patients with DME and elevated OSBP. The improvements in blood pressure and visual acuity, despite fewer injections, indicate a potential synergistic effect of SGLT2i in managing DME with hypertension. Further investigation is warranted to validate its efficacy as a potential systemic adjunct.

**Trial Registration:** UMIN000057674.

## Summary

This post hoc COMET Trial analysis indicates that SGLT2i may lessen intravitreal injection burden and improve blood pressure in patients with diabetic macular edema. Compared to sulfonylurea, SGLT2i demonstrated superior outcomes, suggesting its potential as a systemic adjunct for managing diabetic macular edema with hypertension.

## Graphical abstract

SGLT2i reduced the frequency of intravitreal ranibizumab injections in a subgroup of patients with both diabetic macular edema and hypertension, demonstrating a potential synergistic effect.

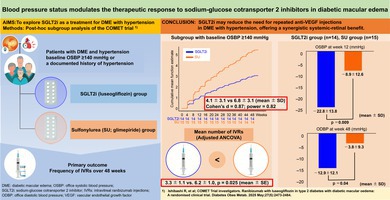

To the Editor,

1

Diabetic macular edema (DME) is frequently associated with poorly controlled cardiovascular risk factors, particularly hypertension. Elevated blood pressure (BP) exacerbates diabetic retinopathy, accelerates retinal arteriolar wall thickening, and increases the risk of DME onset [1]. Sodium‐glucose cotransporter 2 inhibitors (SGLT2i), originally developed for glycemic control, have demonstrated BP reduction and cardiovascular protection [2]. Recent studies suggest that SGLT2i may reduce the burden of intravitreal anti‐vascular endothelial growth factor (VEGF) therapy in DME [3]; however, the influence of baseline BP status on treatment response remains unclear.

## 2 Methods

This post hoc subgroup analysis utilized data from the COMET Trial, a multicenter randomized controlled study evaluating luseogliflozin (SGLT2i) versus glimepiride (SU) in Japanese patients with type 2 diabetes and DME requiring anti‐VEGF therapy [3]. All participants received an initial intravitreal ranibizumab injection (IVR), followed by additional injections based on central retinal thickness (CRT) and best‐corrected visual acuity (BCVA) criteria. Any changes to the antihypertensive medications and diuretics were protocol‐restricted.

Subgroups were defined by baseline office systolic blood pressure (OSBP ≥ 140 mmHg) or documented history of hypertension. The primary endpoint was IVR frequency between Week 0 and Week 48. Missing data were imputed using the last observation carried forward, and post hoc power and adjusted analyses were performed. Secondary endpoints included changes in OSBP, office diastolic blood pressure (ODBP), CRT, and BCVA. Between‐group comparisons used Welch's *t*‐test or *χ* ^2^/Fisher's exact test. Further procedural details are available in the Supporting Information protocol.

## 3 Results

**FIGURE 1:**
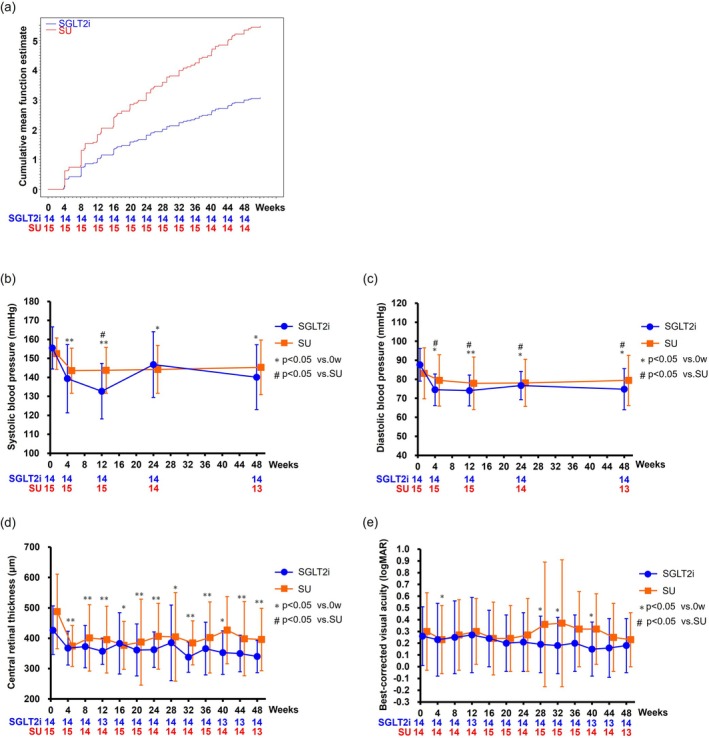
Changes in ophthalmic parameters and blood pressure in patients with baseline OSBP ≥ 140 mmHg. (A) A trend toward fewer cumulative IVRs was observed in the SGLT2i group compared with the SU group. (B) At Week 12, the reduction in OSBP from baseline was significantly greater in the SGLT2i group than in the SU group; however, no significant difference was observed between the groups after Week 24. (C) ODBP was consistently lower in the SGLT2i group compared with the SU group throughout the study period. (D) CRT significantly decreased in both groups, although between‐group differences were not statistically significant. (E) BCVA, measured in logMAR, showed no significant changes in either group. Data in panels B, C, D, and E are expressed as mean ± standard deviation. *p < 0.05 vs. baseline is the *p* value of a paired comparison test with the baseline data within the same group. ^#^ *p* < 0.05 vs. SGLT2i is the *p* value of the between‐group comparison test (Welch's *t*‐test). BCVA, best‐corrected visual acuity; CRT, central retinal thickness; IVR, intravitreal ranibizumab injection; ODBP, office diastolic blood pressure; OSBP, office systolic blood pressure; SGLT2i, sodium‐glucose cotransporter 2 inhibitor; SU, sulfonylurea.

The subgroup with baseline OSBP ≥ 140 mmHg included 14 SGLT2i and 15 SU patients, whereas the subgroup defined by a documented history of hypertension included 18 and 20 patients, respectively (Figure S1). Among patients with baseline OSBP ≥ 140 mmHg, mean age was 64 years, glycated hemoglobin 7.9%, OSBP 153 mmHg, and ODBP 85 mmHg. Approximately half of the patients were receiving antihypertensive treatment, and about half had previously received anti‐VEGF therapy. The SGLT2i group was older and had lower estimated glomerular filtration rate than the SU group. In the subgroup without a documented history of hypertension, body weight was significantly lower in the SGLT2i group than in the SU group (Tables S1 and S2). Among patients with OSBP ≥ 140 mmHg, the total number (mean ± standard deviation) of IVRs administered over the study period, including the initial dose, was 4.1 ± 3.1 and 6.8 ± 3.1 in the SGLT2i and SU groups, respectively, yielding a large effect size (Cohen's *d* = 0.87). A post hoc statistical power of 0.82 supports the robustness of the observed difference. In the adjusted analysis of covariance and Cox models accounting for baseline factors, the treatment group effect remained significant (3.3 ± 1.1 vs. 6.2 ± 1.0, *p* = 0.025). Multiple imputation confirmed a significant between‐group difference in IVR frequency (adjusted Δ −2.7; 95% confidence interval: −5.0 to −0.5; *p* = 0.018). The SGLT2i group tended to receive fewer cumulative IVRs than the SU group (hazard ratio: 0.56, 95% confidence interval: 0.31–1.01; *p* = 0.05) (Figure 1A). SGLT2i treatment led to greater reductions in OSBP at Week 12 (−22.8 ± 13.8 vs. −8.9 ± 12.6 mmHg, *p* = 0.04) and sustained ODBP improvement through Week 48 (−12.9 ± 12.1 vs. −3.8 ± 9.3 mmHg, *p* = 0.04) (Figure 1B,C). In the mediation analyses, indirect effects of BP changes on IVR frequency were not significant (systolic, *p* = 0.40; diastolic, *p* = 0.13). CRT decreased significantly in both groups: from 426.4 ± 79.8 μm to 339.9 ± 53.2 μm in the SGLT2i group (*p* = 0.001) and from 488.1 ± 122.8 μm to 395.7 ± 102.5 μm in the SU group (*p* = 0.013), but intergroup differences were not significant (Figure 1D). BCVA showed a favorable trend in the SGLT2i group, from 0.26 ± 0.25 to 0.18 ± 0.23 by week 48 (*p* = 0.06) (Figure 1E). In contrast, patients with OSBP < 140 mmHg or without hypertension history did not exhibit significant intergroup differences in IVR frequency or BP changes (Figures S2–S4). No significant safety concerns were identified among the groups.

## 4 Comment

In this study, patients were stratified by documented hypertension and elevated OSBP. While some overlap was expected, OSBP may better reflect current hemodynamic status. These findings suggest that elevated OSBP at baseline may influence responsiveness to SGLT2i in DME. As the mediation analyses showed no indirect BP effects on IVR frequency, reduced treatment burden may involve mechanisms beyond BP lowering. In particular, SGLT2i may suppress pericyte degeneration, inhibit microglial activation, and reduce VEGF production, contributing to both vascular and neuroprotective effects [4, 5].

Although BCVA improvement was not statistically significant, stable trends with fewer injections suggest meaningful clinical benefit through reduced burden and preserved vision. The observed reduction in IVR frequency among patients receiving SGLT2i may have implications beyond clinical efficacy, potentially reducing treatment burden, patient visits, and healthcare resource utilization. Although no formal cost‐effectiveness analysis was performed, these findings suggest a favorable impact on real‐world management.

Importantly, the COMET Trial protocol restricted changes in antihypertensive medications, allowing clearer attribution of BP effects to SGLT2i. The reduced administered IVRs suggest a stable fundus status, despite CRT‐guided injection criteria.

These findings highlight the relevance of systemic vascular parameters in DME management. Despite the post hoc design, small subgroups, reliance on office BP measurements, and open‐label nature, IVR differences showed a large effect size and adequate power. The observed responsiveness to SGLT2i in DME with hypertension provides a rationale for future trials stratified by baseline BP to validate therapeutic relevance, assess patient‐centered outcomes, and evaluate long‐term safety and cost‐effectiveness, including the impact of antihypertensive effects on retinal outcomes.

## Author Contributions

All authors meet the authorship criteria established by the International Committee of Medical Journal Editors (ICMJE), affirm responsibility for the integrity of the work, and have approved the final version of the manuscript for publication. R.I. conceived the study and led its design and execution. R.I., M.K., and K.Y. secured research funding. Data collection was performed by R.I., M.K., Y.T., and T.T. The statistical analysis plan was developed by R.I., who also drafted the manuscript. All authors contributed to the interpretation of findings, participated in manuscript revision, and approved the final version. R.I., M.K., Y.T., and T.T. serve as guarantors of the study, with full access to all data and responsibility for its integrity and the accuracy of the analyses.

## Funding

This study was funded by Taisho Pharmaceutical Co. Ltd. The sponsor had no role in the design of the study, data collection and analysis, decision to publish, or preparation of the manuscript.

## Ethics Statement

The COMET Trial was granted ethical approval by the Clinical Research Review Committee of Chiba University Hospital (approval number: 5‐380) and registered with the Japan Registry of Clinical Trials (jRCTs031180210). The Institutional Review Board of Chiba University Graduate School of Medicine approved this study (HK202408‐01), which was registered under UMIN000057674. Participants were offered the opportunity to opt out.

## Consent

All participants provided written informed consent prior to enrolment at the start of the COMET trial. In this post hoc analysis, participants were offered the opportunity to opt out. No patients or members of the public were involved in the design, conduct, or interpretation of this study.

## Conflicts of Interest

R.I. received research grants from Taisho Pharmaceutical Co. Ltd.; Astellas Pharma Inc.; Otsuka Pharmaceutical Co. Ltd.; AstraZeneca K.K.; and Novo Nordisk Pharma Ltd. M.K. and K.Y. received funding from Astellas Pharma Inc. and Taisho Pharmaceutical Co. Ltd., while T.T. and S.Y. received support from Bayer AG. All sponsors had no role in the design, conduct, analysis, or reporting of the study.

## Supporting information


**Figure S1:** CONSORT flow diagram for patient selection.
**Figure S2:** Changes in ophthalmic parameters and blood pressure in patients with baseline OSBP < 140 mmHg.
**Figure S3:** Changes in ophthalmic parameters and blood pressure in patients with a documented history of hypertension.
**Figure S4:** Changes in ophthalmic parameters and blood pressure in patients without a documented history of hypertension.
**Table S1:** Baseline characteristics of patients stratified by office systolic blood pressure.
**Table S2:** Baseline characteristics of patients stratified by a documented history of hypertension.

**Data S1:** jdb70184‐sup‐0002‐Supinfo2.docx.

## Data Availability

The data that support the findings of this study are available on request from the corresponding author. The data are not publicly available due to privacy or ethical restrictions.
