# Supplementary material for: Blood Pressure Status Modulates the Therapeutic Response to Sodium‐Glucose Cotransporter 2 Inhibitors in Diabetic Macular Edema: A Post Hoc Subgroup Analysis of the COMET Trial
Source: J Diabetes. 2025 Dec 18;17(12):e70184. doi: 10.1111/1753-0407.70184 (PMC12715334; doi:10.1111/1753-0407.70184)
Supplement: Supplementary file 1 — Figure S1: CONSORT flow diagram for patient selection. Figure S2: Changes in ophthalmic parameters and blood pressure in patients with baseline OSBP < 140 mmHg. Figure S3: Changes in ophthalmic parameters and blood pressure in patients with a documented history of hypertension. Figure S4: Changes in ophthalmic parameters and blood pressure in patients without a documented history of hypertension. Table S1: Baseline characteristics of patients stratified by office systolic blood pressure. Table S2: Baseline characteristics of patients stratified by a documented history of hypertension. [file JDB-17-e70184-s001.docx]

**Supplementary Material**

Supplemental Figure S1. CONSORT flow diagram for patient selection

Supplemental Figure S2. Changes in ophthalmic parameters and blood pressure in patients with baseline OSBP <140 mmHg

Supplemental Figure S3. Changes in ophthalmic parameters and blood pressure in patients with a documented history of hypertension

Supplemental Figure S4. Changes in ophthalmic parameters and blood pressure in patients without a documented history of hypertension

Supplemental Table S1. Baseline characteristics of patients stratified by office systolic blood pressure

Supplemental Table S2. Baseline characteristics of patients stratified by a documented history of hypertension

Supplemental Figure 1. CONSORT flow diagram for patient selection


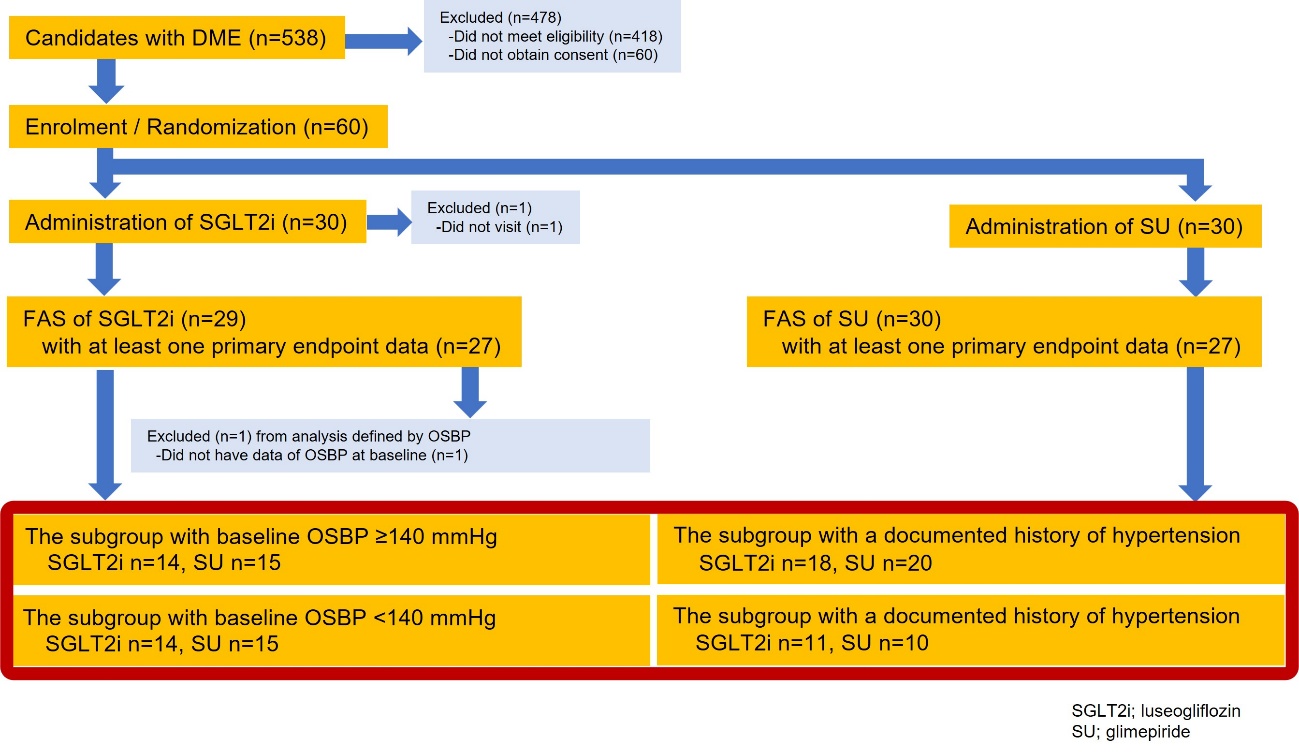


Using the FAS dataset obtained from the COMET trial, patients were divided into four groups based on OSBP and a documented history of hypertension. The FAS of the COMET Trial comprised 29 patients in the SGLT2i group and 30 patients in the SU group. One patient in the SGLT2i group discontinued follow-up visits. All 59 patients received at least one additional anti-VEGF treatment. Endpoint data through 48 weeks were available for 27 patients in each group. In the SGLT2i group, one patient was excluded from the subgroup analysis of OSBP due to missing baseline (week 0) OSBP data. The subgroup with a baseline OSBP ≥140 mmHg included 14 patients in the SGLT2i group and 15 in the SU group, whereas the subgroup defined by a documented history of hypertension included 18 and 20 patients, respectively.

CONSORT, CONsolidated Standards Of Reporting Trials flow diagram; DME, diabetic macular edema; FAS, full analysis set; OSBP, office systolic blood pressure; SGLT2i, sodium-glucose cotransporter 2 inhibitor; SU, sulfonylurea

**Supplemental Figure S2. Changes in ophthalmic parameters and blood pressure in patients with baseline OSBP <140 mmHg**


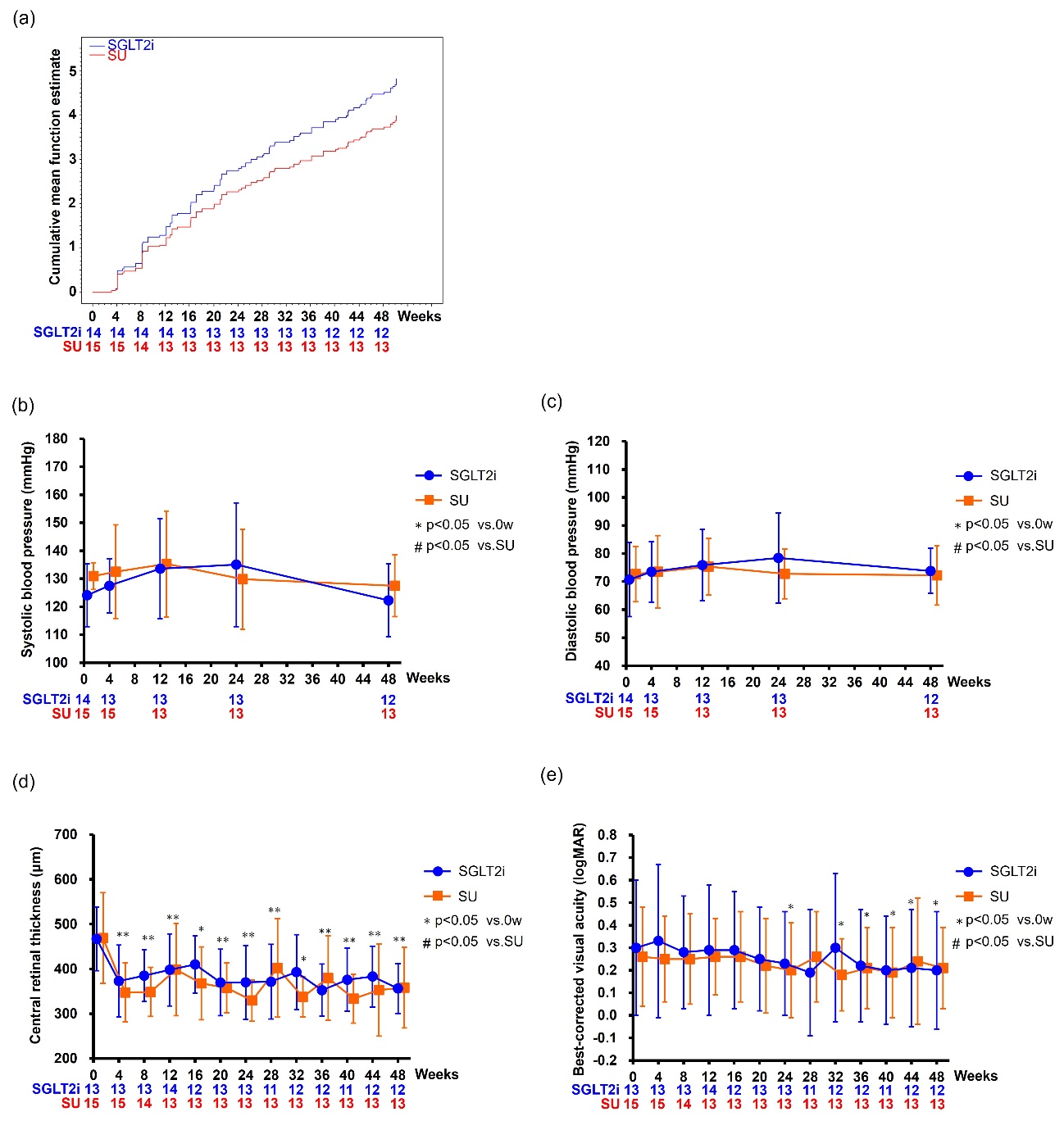


The cumulative mean number of IVRs is shown in Panel a. OSBP, ODBP, CRT, and BCVA changes are shown in panels b, c, d, and e, respectively. No significant differences were observed between the two groups. These panels show mean ± standard deviation. * p<0.05 vs. 0w is the p value of a paired comparison test with the baseline data within the same group. # p<0.05 vs. SGLT2i is the p value of the intergroup comparison test.

The total number of IVRs administered over the 48-week study period, including the initial dose, was 6.1 ± 3.8 in the SGLT2i group and 4.9 ± 2.1 in the SU group (mean ± SD). Analysis of covariance, adjusting for allocation factors, revealed no significant difference in the mean number of IVRs between the two groups (SGLT2i group: 5.8 ± 1.3; SU group: 4.9 ± 1.1; mean ± SE, p = 0.54).

The cumulative number of IVRs following intervention did not differ significantly between groups (HR, 1.21; 95% CI, 0.76–1.94; p = 0.43) (Supplemental Fig. S2a).

Neither OSBP nor ODBP showed significant changes over the 48-week period, and no differences were observed between the groups (Supplemental Fig. S2b and S2c).

CRT significantly decreased in both groups: from 467.3 ± 71.1 μm to 356.3 ± 55.2 μm in the SGLT2i group (p = 0.005), and from 469.1 ± 101.4 μm to 358.2 ± 90.0 μm in the SU group (p = 0.001). However, no significant intergroup differences were noted (Supplemental Fig. S2d).

BCVA, expressed in logMAR, showed significant improvement in the SGLT2i group (0.30 ± 0.30 to 0.20 ± 0.26; p = 0.009). In contrast, the SU group showed no statistically significant improvement (0.26 ± 0.22 to 0.21 ± 0.18; p = 0.07) at week 48. However, no significant intergroup differences were noted at week 48 (Supplemental Fig. S2e).

BCVA, best-corrected visual acuity; CRT, central retinal thickness; IVR, intravitreal injection of ranibizumab; ODBP, office diastolic blood pressure; OSBP, office systolic blood pressure; SGLT2i, sodium-glucose cotransporter 2 inhibitors; SU, sulfonylurea

**Supplemental Figure S3. Changes in ophthalmic parameters and blood pressure in patients with a documented history of hypertension**

**
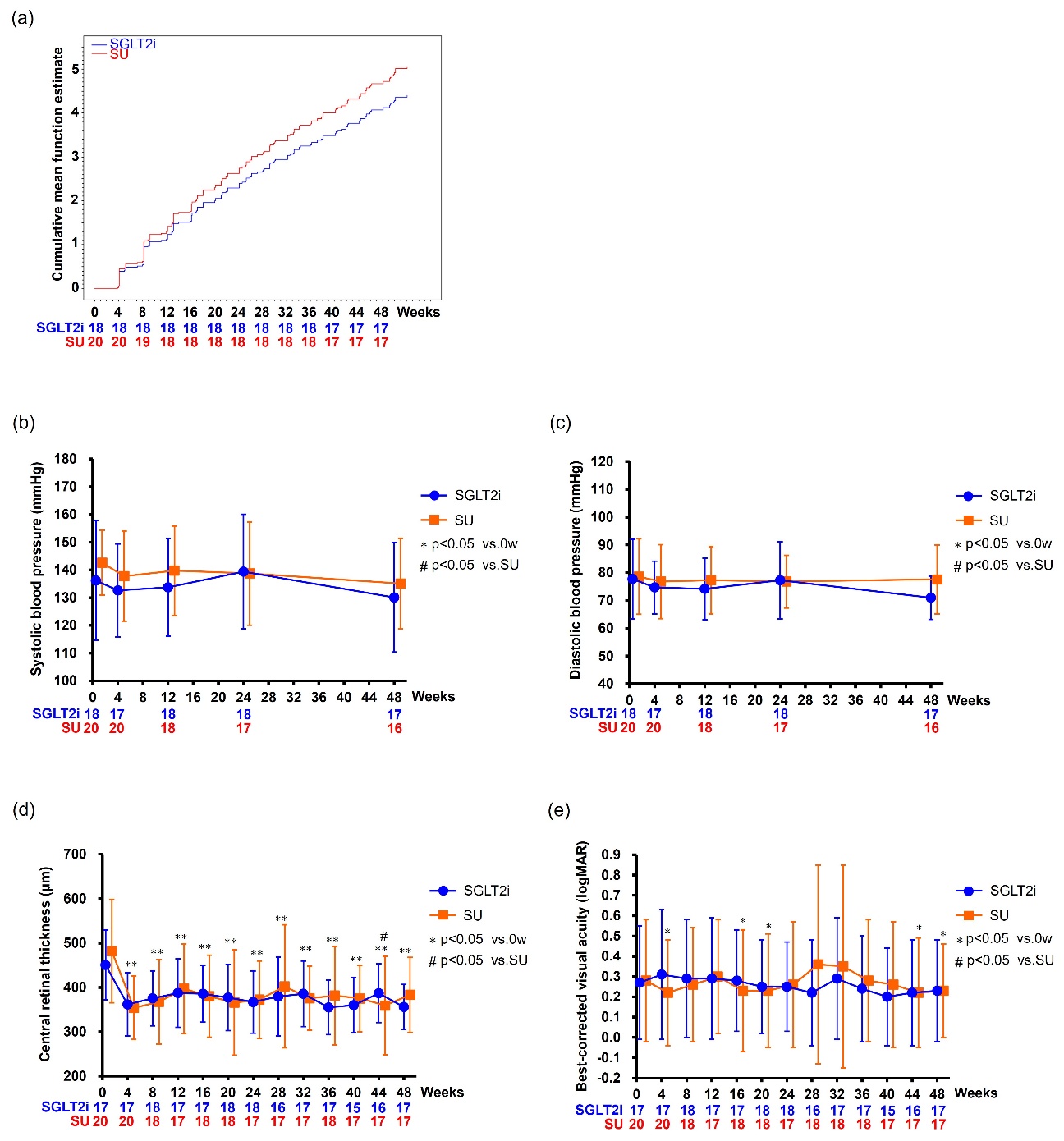
**

The cumulative mean number of IVRs is shown in panel a. OSBP, ODBP, CRT, and BCVA changes are shown in panels b, c, d, and e, respectively. No significant differences were observed between the two groups. These panels show mean ± standard deviation. * p<0.05 vs. 0w is the p value of a paired comparison test with the baseline data within the same group. # p<0.05 vs. SGLT2i is the p value of the intergroup comparison test.

The total number of IVRs administered over the 48-week study period, including the initial dose, was 5.5 ± 3.8 in the SGLT2i group and 6.2 ± 3.1 in the SU group (mean ± SD). Analysis of covariance, adjusted for allocation factors, revealed no significant difference in the mean number of IVRs between groups (SGLT2i group: 5.4 ± 1.0; SU group: 6.0 ± 1.0; mean ± SE, p = 0.64).

The cumulative IVR frequency did not differ significantly between groups (HR, 0.87; 95% CI, 0.54–1.40; p = 0.57) (Supplemental Fig. S3a).

Neither OSBP nor ODBP changed significantly over the 48-week period, and no differences were observed between the groups (Supplemental Fig. S3b and S3c).

CRT significantly decreased in both groups: from 450.9 ± 78.5 μm to 356.1 ± 50.6 μm in the SGLT2i group (p = 0.005), and from 481.8 ± 116.6 μm to 383.2 ± 84.6 μm in the SU group (p < 0.001). However, no significant intergroup differences were observed at week 48 (Supplemental Fig. S3d).

BCVA, expressed in logMAR, improved significantly in the SU group (0.28 ± 0.30 to 0.23 ± 0.23; p = 0.025). In contrast, the SGLT2i group showed no statistically significant improvement (0.27 ± 0.28 to 0.23 ± 0.25; p = 0.07). Nonetheless, no significant differences between the two groups were found at week 48 (Supplemental Fig. S3e).

BCVA, best-corrected visual acuity; CRT, central retinal thickness; IVR, intravitreal injection of ranibizumab; ODBP, office diastolic blood pressure; OSBP, office systolic blood pressure; SGLT2i, sodium-glucose cotransporter 2 inhibitors; SU, sulfonylurea

**Supplemental Figure S4. Changes in ophthalmic parameters and blood pressure in patients without a documented history of hypertension**


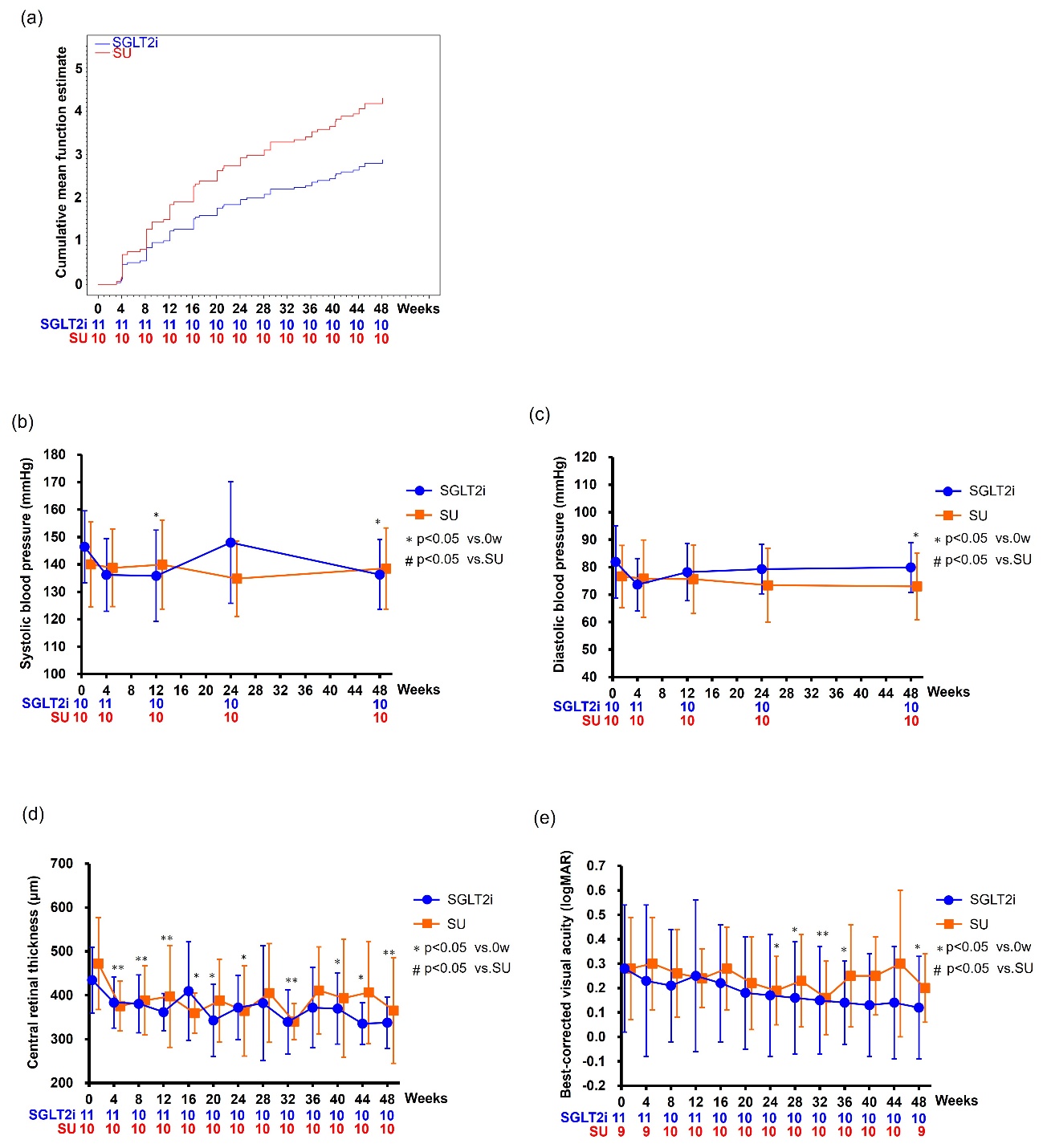


The cumulative mean number of IVRs is shown in panel a. OSBP, ODBP, CRT, and BCVA changes are shown in panels b, c, d, and e, respectively. No significant differences were observed between the two groups. These panels show mean ± standard deviation. * p<0.05 vs. 0w is the p value of a paired comparison test with the baseline data within the same group. # p<0.05 vs. SGLT2i is the p value of the intergroup comparison test.

The total number of IVRs administered over the 48-week study period, including the initial dose, was 3.9 ± 2.6 in the SGLT2i group and 5.3 ± 2.2 in the SU group (mean ± SD). Analysis of covariance, adjusted for allocation factors, revealed no significant difference in the mean number of IVRs between the two groups (SGLT2i group: 3.7 ± 1.0; SU group: 4.8 ± 1.3; mean ± SE, p = 0.36).

The cumulative IVR frequency did not differ significantly between groups (HR, 0.67; 95% CI, 0.37–1.21; p = 0.18) (Supplemental Fig. S4a).

At 48 weeks, OSBP was significantly reduced in the SGLT2i group (−12.2 ± 11.3 mmHg; p = 0.012), but not in the SU group (−1.5 ± 12.9 mmHg; p = 0.72). However, there was no significant difference in OSBP between the two groups at week 48 (Supplemental Fig. S4b). ODBP was significantly reduced in the SU group at week 48 (−3.6 ± 4.9 mmHg; p = 0.046), but not in the SGLT2i group (−3.7 ± 12.6 mmHg; p = 0.41). No significant intergroup difference in ODBP was observed (Supplemental Fig. S4c).

CRT significantly decreased in both groups: from 434.7 ± 75.0 μm to 337.3 ± 58.6 μm in the SGLT2i group (p = 0.002), and from 472.3 ± 104.7 μm to 365.1 ± 120.3 μm in the SU group (p = 0.019). However, intergroup differences were not significant (Supplemental Fig. S4d).

BCVA, expressed in logMAR, showed significant improvement in the SGLT2i group (0.28 ± 0.26 to 0.12 ± 0.21; p = 0.024). In contrast, the SU group showed no statistically significant improvement (0.28 ± 0.21 to 0.20 ± 0.14; p = 0.69) at week 48. However, no significant intergroup differences were noted at week 48 (Supplemental Fig. S4e).

BCVA, best-corrected visual acuity; CRT, central retinal thickness; IVR, intravitreal injection of ranibizumab; ODBP, office diastolic blood pressure; OSBP, office systolic blood pressure; SGLT2i, sodium-glucose cotransporter 2 inhibitors; SU, sulfonylurea

**Supplemental Table S1. Baseline characteristics of patients stratified by office systolic blood pressure**

|  | OSBP ≥140 mmHg | | | OSBP <140 mmHg | | |
| --- | --- | --- | --- | --- | --- | --- |
|  | SGLT2i | SU | p-value | SGLT2i | SU | p-value |
| N | 14 | 15 |  | 14 | 15 |  |
| Age (years) | 68.1±7.6 | 60.8±11.2 | 0.048 | 60.8±11.5 | 64.1±7.7 | 0.38 |
| Sex |  |  | 0.45 |  |  | 1.00 |
| Male | 10 | 8 |  | 9 | 10 |  |
| Female | 4 | 7 |  | 5 | 5 |  |
| Diabetes | 14 | 15 |  | 14 | 15 |  |
| Duration of diabetes (years) | 16.4±9.5 | 12.5±12.3 | 0.36 | 13.9±11.4 | 12.8±9.3 | 0.79 |
| Treatment for diabetes |  |  |  |  |  |  |
| Insulin | 5 | 2 | 0.21 | 6 | 6 | 1.00 |
| GLP-1 RA | 0 | 0 | - | 2 | 3 | 1.00 |
| DPP4i | 13 | 12 | 0.60 | 8 | 10 | 0.71 |
| Metformin | 10 | 12 | 0.68 | 10 | 12 | 0.68 |
| Thiazolidine | 0 | 0 | - | 0 | 1 | 1.00 |
| Glinide | 0 | 1 | 1.00 | 1 | 0 | 0.48 |
| Alpha GI | 4 | 4 | 1.00 | 7 | 3 | 0.13 |
| Hypertension | 7 | 10 | 0.46 | 11 | 10 | 0.68 |
| Antihypertensive | 7 | 9 | 0.72 | 10 | 7 | 0.26 |
| RAS inhibitors | 6 | 7 | 1.00 | 8 | 6 | 0.47 |
| Diuretic | 1 | 0 | 0.48 | 2 | 1 | 0.60 |
| Dyslipidemia | 9 | 10 | 1.00 | 11 | 10 | 0.68 |
| Lipid-lowering | 9 | 9 | 1.00 | 10 | 7 | 0.26 |
| Cardiovascular disease |  |  |  |  |  |  |
| Stroke | 0 | 0 | - | 0 | 0 | - |
| Myocardial infarction | 0 | 0 | - | 0 | 0 | - |
| Chronic heart failure | 1 | 1 | 1.00 | 1 | 0 | 0.48 |
| Chronic kidney disease | 0 | 0 | - | 2 | 1 | 0.60 |
| Antiplatelet | 1 | 0 | 0.48 | 3 | 0 | 0.10 |
| Anticoagulant | 2 | 0 | 0.22 | 1 | 1 | 1.00 |
| Diabetes eye |  |  |  |  |  |  |
| Diabetic retinopathy | 14 | 15 | 1.00 | 13 | 15 | 0.15 |
| Non-proliferative retinopathy | 13 | 13 |  | 9 | 14 |  |
| Proliferative retinopathy | 1 | 2 |  | 4 | 1 |  |
| Treatment |  |  |  |  |  |  |
| Panretinal photocoagulation | 4 | 6 | 0.70 | 7 | 3 | 0.13 |
| Diabetic macular edema |  |  |  |  |  |  |
| Target eye | 14 | 15 | - | 13 | 15 | - |
| Spongy edema | 11 | 11 | 1.00 | 12 | 9 | 0.08 |
| Cystoid edema | 9 | 8 | 0.71 | 8 | 10 | 1.00 |
| Serous detachment | 1 | 2 | 1.00 | 2 | 7 | 0.11 |
| Fellow eye | 11 | 9 | 0.68 | 9 | 10 | 1.00 |
| Spongy edema | 8 | 8 | 0.59 | 7 | 5 | 0.35 |
| Cystoid edema | 3 | 3 | 1.00 | 2 | 6 | 0.17 |
| Serous detachment | 0 | 2 | 0.19 | 3 | 1 | 0.30 |
| Treatment |  |  |  |  |  |  |
| Anti-VEGF agent | 7 | 9 | 0.72 | 7 | 7 | 1.00 |
| Steroid (triamcinolone acetonide) |  |  |  |  |  |  |
| Sub-Tenon's | 0 | 4 | 0.10 | 7 | 2 | 0.05 |
| Intravitreal | 1 | 1 | 1.00 | 0 | 0 | - |
| Retinal arteriole aneurysm direct photocoagulation | 3 | 5 | 0.68 | 1 | 5 | 0.17 |
| Macular subthreshold laser | 1 | 1 | 1.00 | 0 | 0 | - |
| Photocoagulation | 6 | 9 | 0.47 | 7 | 5 | 0.46 |
| Cataract | 7 | 8 | 1.00 | 8 | 11 | 0.45 |
| Post-surgery | 7 | 4 | 0.26 | 4 | 5 | 1.00 |
| Glaucoma | 1 | 0 | 0.48 | 1 | 1 | 1.00 |
| Under eye drop treatment | 1 | 0 | 0.48 | 0 | 0 | - |
| BMI (kg/m^2^) | 24.2±2.4 | 25.7±3.9 | 0.23 | 24.2±4.0 | 25.7±3.9 | 0.31 |
| OSBP (mmHg) | 155.5±11.1 | 152.5±8.3 | 0.43 | 124.1±11.2 | 130.9±4.8 | 0.05 |
| ODBP (mmHg) | 87.6±8.6 | 83.1±13.4 | 0.28 | 70.7±13.2 | 72.7±9.9 | 0.65 |
| Pulse (bpm) | 71.4±12.5 | 77.3±17.1 | 0.37 | 84.7±11.0 | 79.7±15.9 | 0.44 |
| HbA1c (%) | 8.1±1.2 | 7.7±1.3 | 0.30 | 7.3±0.6 | 7.7±0.8 | 0.16 |
| Fasting BG (mg/dL) | 182.1±42.6 | 176.9±47.6 | 0.77 | 141.9±20.7 | 158.6±47.4 | 0.23 |
| eGFR (mL/min/1.73m^2^) | 66.6±10.5 | 80.9±14.1 | <0.01 | 69.1±21.9 | 73.6±21.1 | 0.57 |
| U-Alb (mg/g.Cre) | 197.2±340.9 | 217.4±321.3 |  | 544.6±965.7 | 216.4±354.5 |  |
| (Logarithmic conversion value) | 3.99±1.84 | 4.43±1.42 | 0.48 | 4.46±2.16 | 4.39±1.47 | 0.92 |
| Hct (%) | 42.9±3.8 | 40.4±3.8 | 0.09 | 39.6±3.7 | 41.6±3.1 | 0.12 |
| AST (IU/L) | 29.6±13.5 | 22.6±6.3 | 0.09 | 20.2±4.4 | 24.7±7.6 | 0.06 |
| ALT (IU/L) | 31.8±14.1 | 28.1±15.2 | 0.51 | 19.1±8.2 | 25.2±9.8 | 0.08 |
| γGTP (IU/L) | 33.9±24.4 | 34.5±23.2 |  | 34.9±53.5 | 31.3±24.2 |  |
| (Logarithmic conversion value) | 3.32±0.65 | 3.38±0.56 | 0.80 | 3.14±0.74 | 3.26±0.57 | 0.62 |

Patient characteristics were compared using Pearson’s chi-square or Fisher’s exact test for categorical endpoints and Welch’s t-test for continuous variables. Data are presented as mean ± standard deviation. Data were compared between two groups using the one-sample *t*-test. Urinary albumin levels and γGTP were compared using the logarithmic conversion value.

alpha GI, alpha glucosidase inhibitor; ALT, alanine aminotransferase; AST, aspartate aminotransferase; BG, blood glucose; BMI, body mass index; DPP4i, dipeptidyl peptidase-4 inhibitor; eGFR, estimated glomerular filtration rate; GLP-1 RA, glucagon-like peptide-1 receptor agonist; GTP, glutamyl transpeptidase; HbA1c, glycated hemoglobin; Hct, hematocrit; ODBP, office diastolic blood pressure; OSBP, office systolic blood pressure; RAS, renin-angiotensin system; SGLT2i, sodium-glucose cotransporter 2 inhibitor; SU, sulfonylurea; U-Alb, urinary albumin; VEGF, vascular endothelial growth factor

**Supplemental Table S2. Baseline characteristics of patients stratified by a documented history of hypertension**

|  | Hypertension (+) | | | Hypertension (-) | | |
| --- | --- | --- | --- | --- | --- | --- |
|  | SGLT2i | SU | p-value | SGLT2i | SU | p-value |
| N | 18 | 20 |  | 11 | 10 |  |
| Age (years) | 63.8±9.4 | 61.1±10.2 | 0.40 | 65.9±11.5 | 65.2±7.9 | 0.87 |
| Sex |  |  | 1.00 |  |  | 0.15 |
| Male | 10 | 12 |  | 10 | 6 |  |
| Female | 8 | 8 |  | 1 | 4 |  |
| Diabetes | 18 | 20 |  | 11 | 10 |  |
| Duration of diabetes (years) | 15.6±9.8 | 11.8±11.8 | 0.31 | 13.6±11.4 | 14.1±9.0 | 0.92 |
| Treatment for diabetes |  |  |  |  |  |  |
| Insulin | 7 | 5 | 0.49 | 4 | 3 | 1.00 |
| GLP-1 RA | 2 | 1 | 0.59 | 0 | 2 | 0.21 |
| DPP4i | 13 | 15 | 1.00 | 9 | 7 | 0.64 |
| Metformin | 12 | 15 | 0.72 | 9 | 9 | 1.00 |
| Thiazolidine | 0 | 1 | 1.00 | 0 | 0 | - |
| Glinide | 1 | 0 | 0.47 | 0 | 1 | 0.48 |
| Alpha GI | 8 | 4 | 0.16 | 3 | 3 | 1.00 |
| Hypertension | 18 | 20 | - | 0 | 0 | - |
| Antihypertensive | 17 | 16 | 0.34 | 0 | 0 | - |
| RAS inhibitors | 14 | 13 | 0.48 | 0 | 0 | - |
| Diuretic | 3 | 1 | 0.33 | 0 | 0 | - |
| Dyslipidemia | 15 | 13 | 0.28 | 5 | 7 | 0.39 |
| Lipid-lowering | 14 | 10 | 0.10 | 5 | 6 | 0.67 |
| Cardiovascular disease |  |  |  |  |  |  |
| Stroke | 0 | 0 | - | 0 | 0 | - |
| Myocardial infarction | 0 | 0 | - | 0 | 0 | - |
| Chronic heart failure | 2 | 1 | 0.59 | 0 | 0 | - |
| Chronic kidney disease | 2 | 1 | 0.59 | 0 | 0 | - |
| Antiplatelet | 3 | 0 | 0.10 | 2 | 0 | 0.48 |
| Anticoagulant | 2 | 0 | 0.22 | 1 | 1 | 1.00 |
| Diabetes eye |  |  |  |  |  |  |
| Diabetic retinopathy | 17 | 20 | 0.16 | 11 | 10 | 1.00 |
| Non-proliferative retinopathy | 13 | 19 |  | 9 | 8 |  |
| Proliferative retinopathy | 4 | 1 |  | 2 | 2 |  |
| Treatment |  |  |  |  |  |  |
| Panretinal photocoagulation | 8 | 8 | 1.00 | 4 | 1 | 0.31 |
| Diabetic macular edema |  |  |  |  |  |  |
| Target eye | 17 | 20 | - | 11 | 10 | - |
| Spongy edema | 14 | 15 | 0.70 | 9 | 5 | 0.18 |
| Cystoid edema | 13 | 11 | 0.30 | 5 | 7 | 0.39 |
| Serous detachment | 1 | 6 | 0.10 | 2 | 3 | 0.64 |
| Fellow eye | 13 | 15 | 1.00 | 8 | 4 | 0.63 |
| Spongy edema | 9 | 11 | 1.00 | 6 | 2 | 0.55 |
| Cystoid edema | 4 | 6 | 0.71 | 2 | 3 | 0.22 |
| Serous detachment | 3 | 2 | 0.64 | 0 | 1 | 0.33 |
| Treatment |  |  |  |  |  |  |
| Anti-VEGF agent | 10 | 10 | 0.76 | 5 | 6 | 0.67 |
| Steroid (triamcinolone acetonide) |  |  |  |  |  |  |
| Sub-Tenon's | 6 | 3 | 0.26 | 2 | 3 | 0.64 |
| Intravitreal | 1 | 0 | 0.47 | 0 | 1 | 0.48 |
| Retinal arteriole aneurysm direct photocoagulation | 3 | 5 | 0.70 | 2 | 5 | 0.18 |
| Macular subthreshold laser | 1 | 0 | 0.47 | 0 | 1 | 0.48 |
| Photocoagulation | 9 | 12 | 0.74 | 5 | 2 | 0.36 |
| Cataract | 9 | 14 | 0.32 | 7 | 5 | 0.67 |
| Post-surgery | 8 | 5 | 0.31 | 3 | 4 | 0.66 |
| Glaucoma | 1 | 1 | 1.00 | 1 | 0 | 1.00 |
| Under eye drop treatment | 1 | 0 | 0.47 | 0 | 0 | - |
| BMI (kg/m^2^) | 24.9±3.5 | 25.6±4.2 | 0.57 | 22.8±2.4 | 25.9±3.1 | 0.03 |
| OSBP (mmHg) | 136.2±21.6 | 142.6±11.7 | 0.27 | 146.4±13.1 | 140.0±15.5 | 0.33 |
| ODBP (mmHg) | 77.7±14.4 | 78.6±13.6 | 0.85 | 81.9±13.1 | 76.6±11.3 | 0.35 |
| Pulse (bpm) | 77.4±12.7 | 80.5±17.4 | 0.60 | 76.3±16.4 | 75.0±14.4 | 0.88 |
| HbA1c (%) | 7.8±1.1 | 7.7±1.1 | 0.67 | 7.5±0.7 | 7.7±0.9 | 0.70 |
| Fasting BG (mg/dL) | 164.4±40.9 | 162.6±47.7 | 0.91 | 157.8±36.0 | 173.7±48.9 | 0.42 |
| eGFR (mL/min/1.73m^2^) | 66.0±18.0 | 73.8±17.7 | 0.19 | 71.1±15.1 | 84.3±17.4 | 0.09 |
| U-Alb (mg/g.Cre) | 405.5±836.0 | 255.5±345.3 |  | 308.6±531.1 | 139.6±307.3 |  |
| (Logarithmic conversion value) | 4.36±1.89 | 4.72±1.39 | 0.51 | 3.98±2.22 | 3.79±1.34 | 0.82 |
| Hct (%) | 40.8±3.6 | 40.6±3.7 | 0.91 | 42.1±4.8 | 41.8±3.1 | 0.89 |
| AST (IU/L) | 23.8±10.7 | 22.7±6.9 | 0.72 | 26.9±11.6 | 25.6±6.9 | 0.76 |
| ALT (IU/L) | 24.9±14.2 | 26.1±13.9 | 0.79 | 26.4±11.2 | 27.8±10.1 | 0.77 |
| γGTP (IU/L) | 37.2±48.4 | 31.9±20.6 |  | 29.2±22.9 | 35.0±29.2 |  |
| (Logarithmic conversion value) | 3.25±0.74 | 3.31±0.55 | 0.81 | 3.18±0.62 | 3.35±0.62 | 0.56 |

Patient characteristics were compared using Pearson’s chi-square or Fisher’s exact test for categorical endpoints and Welch’s t-test for continuous variables. Data are presented as mean ± standard deviation. Data were compared between two groups using the one-sample *t*-test. Urinary albumin levels and γGTP were compared using the logarithmic conversion value.

alpha GI, alpha glucosidase inhibitor; ALT, alanine aminotransferase; AST, aspartate aminotransferase; BG, blood glucose; BMI, body mass index; DPP4i, dipeptidyl peptidase-4 inhibitor; eGFR, estimated glomerular filtration rate; GLP-1 RA, glucagon-like peptide-1 receptor agonist; GTP, glutamyl transpeptidase; HbA1c, glycated hemoglobin; Hct, hematocrit; ODBP, office diastolic blood pressure; OSBP, office systolic blood pressure; RAS, renin-angiotensin system; SGLT2i, sodium-glucose cotransporter 2 inhibitor; SU, sulfonylurea; U-Alb, urinary albumin; VEGF, vascular endothelial growth factor
