# Supplementary material for: Blood Pressure Status Modulates the Therapeutic Response to Sodium‐Glucose Cotransporter 2 Inhibitors in Diabetic Macular Edema: A Post Hoc Subgroup Analysis of the COMET Trial
Source: J Diabetes. 2025 Dec 18;17(12):e70184. doi: 10.1111/1753-0407.70184 (PMC12715334; doi:10.1111/1753-0407.70184)
Supplement: Supplementary file 2 — Data S1: jdb70184‐sup‐0002‐Supinfo2.docx. [file JDB-17-e70184-s002.docx]

**Trial Protocol** **and Statistical Analysis Plan**

*Significance and Scientific Rationale*

Patients with diabetic macular edema (DME) frequently exhibit poorly controlled cardiovascular risk factors, which are known to worsen clinical outcomes and exacerbate both diabetic retinopathy (DR) and DME. Among these, hypertension is recognized as a key driver of disease progression, and stringent blood pressure control has been shown to reduce the incidence of proliferative DR and DME.

Sodium–glucose cotransporter 2 inhibitors (SGLT2i), originally developed as antihyperglycemic agents, have since demonstrated a range of systemic metabolic benefits, including reductions in blood pressure. However, no study to date has specifically examined the effect of their antihypertensive properties on DME progression or evaluated treatment outcomes stratified by hypertensive status.

*Study Objective*

Using data from the COMET trial, titled *“Effectiveness and safety of combination therapy of ranibizumab and luseogliflozin in type 2 diabetes with diabetic macular edema: a parallel group comparison with standard treatment controlled by glimepiride”*,¹^,^² we aimed to evaluate the feasibility and efficacy of SGLT2i as a non-invasive treatment option for DME in patients with hypertension. This was achieved through post hoc subgroup analyses comparing outcomes between participants with and without hypertension.

*Compliance with Ethical Guidelines*

The COMET trial was approved by Chiba University Hospital's clinical research review committee (approval number: 5-380) and registered in the University Hospital Medical Information Network Clinical Trial Registry (UMIN000033961) and the Japan Registry of Clinical Trials (jRCTs031180210). Written informed consent was obtained from all participants at the time of the COMET trial enrolment. Participants did not receive any compensation. This study, a post-hoc analysis of the COMET trial, was approved by the Institutional Review Board of the Chiba University Graduates School of Medicine (HK202408-01 approved 25 September 2024) and registered in the University Hospital Medical Information Network Clinical Trial Registry (UMIN000057674).

*Study Design*

The COMET Trial began in April 2018 and concluded in September 2023. Patient enrolment started in April 2019 and was completed by September 2023. Patients eligible for anti-vascular endothelial growth factor (VEGF) therapy were invited to participate, with informed consent obtained before the screening test. Those meeting the eligibility criteria were assigned to either the study agent treatment group (SGLT2i group) or the control group (sulfonylurea [SU] group) (Figure 1). The inclusion and exclusion criteria are shown in Table 1. The follow-up details are described in Table 2.

This post-hoc subgroup analysis focused on DME with hypertension cases based on data from the COMET Trial. The trial’s protocol and results have been previously reported.^1,2^ The study included patients with type 2 diabetes and DME eligible for anti-VEGF therapy, randomized at a 1:1 ratio into two groups: the SGLT2i group, receiving luseogliflozin as the study agent, and the SU group, receiving glimepiride as the control agent. Allocation factors included glycated hemoglobin, urinary albumin, and best-corrected visual acuity (BCVA).

All participants received an initial intravitreal ranibizumab (IVR) injection, followed by additional IVR administrations every four weeks for up to 48 weeks, according to the following criteria:

- Optical coherence tomography (OCT)-measured central retinal thickness (CRT) >350 µm

- OCT-measured CRT increase of >100 µm from the post-enrolment reference CRT

- OCT-measured CRT increase relative to the previous observation point and BCVA increase of 0.2 logMAR from post-enrolment

For patients with bilateral DME, the more severely affected eye was designated as the study eye. For this analysis, patients were classified into subgroups based on baseline office systolic blood pressure (OSBP ≥140 mmHg) or a documented history of hypertension. Comparisons were also conducted with patients who did not meet either criterion.

*Outcomes*

The primary outcome was the difference in the number of IVR between the groups from week 4 to week 48. Secondary outcomes included changes in OSBP, office diastolic blood pressure (ODBP), CRT, and BCVA.

*Data Collection and Analysis*

The full analysis set (FAS) derived from the COMET Trial included the following variables: age, sex, findings from physical examinations, medical and ophthalmologic history, treatment history, procedures performed during the study period, and longitudinal blood, urine, and fundus imaging data. Primary and secondary outcomes were assessed using the FAS, with exclusion of patients who had severe protocol violations.

Baseline characteristics are summarized as frequencies and proportions for categorical variables and as means and standard deviations for continuous variables. Categorical variables were compared using Pearson’s chi-square test or Fisher’s exact test, as appropriate; continuous variables were analyzed using Welch’s t-test. Missing data due to non-attendance were imputed using the last observation carried forward (LOCF) method.

Cox regression analysis was employed to evaluate the number of IVRs as recurrent events following initiation of the intervention. Additionally, IVR re-administration rates were analyzed using the Cox proportional hazards model and visualized as cumulative event-rate curves based on the Kaplan–Meier method. A two-sided p-value <0.05 was considered statistically significant. All statistical analyses were conducted by an independent third-party organization using SAS software version 9.4 (SAS Institute, Cary, NC), under the supervision of certified biostatisticians.

Post-hoc power analysis was performed using G*Power (version 3.1.9.7, Heinrich Heine University Düsseldorf) to estimate achieved power based on group means and standard deviations from comparisons with statistically significant differences.

*Additional Analyses*

Analysis of covariance (ANCOVA) and Cox models were constructed, adjusting for duration of diabetes, baseline CRT, age, and baseline estimated glomerular infiltration rate. Mediation analyses were performed to examine whether blood pressure changes mediated the number of interventions. Sensitivity analyses using multiple imputation with ANCOVA and Cox models were also performed to confirm robustness.

*Role of the Funding Source*

This study received funding from Taisho Pharmaceutical Co., Ltd., which did not influence the study design, data collection and analysis, publication decision, or manuscript preparation.

*Study Implementation Framework*

The COMET Trial was conducted by the members shown in Table 3. This study was conducted by the researchers listed below.

- Principal Investigator: Koutaro Yokote, Department of Endocrinology, Haematology and Gerontology, Chiba University Graduate School of Medicine.
- Secondary Sponsor: Shuichi Yamamoto, Department of Ophthalmology and Vision Science, Chiba University Graduate School of Medicine.
- Research Administration Office Representatives: Ryoichi Ishibashi, Department of Medicine, Division of Diabetes, Endocrinology and Metabolism, Kimitsu Chuo Hospital. Masaya Koshizaka, Centre for Preventive Medical Sciences, Chiba University.
- Lead researcher of this study: Ryoichi Ishibashi, Department of Medicine, Division of Diabetes, Endocrinology and Metabolism, Kimitsu Chuo Hospital.
- Statistical Analysis Manager: Hirokazu Yamada, EviPro Inc. Statistics and Systems Group.

*Research Coordination Committee*

The following members were responsible for planning the study and coordinating its execution:

- Ryoichi Ishibashi, Department of Medicine, Division of Diabetes, Endocrinology and Metabolism, Kimitsu Chuo Hospital
- Masaya Koshizaka, Centre for Preventive Medical Sciences, Chiba University.
- Yoko Takatsuna, Department of Ophthalmology, Chiba Rosai Hospital
- Tomoaki Tatsumi, Department of Ophthalmology and Vision Science, Chiba University Graduate School of Medicine.

**Appendix**

Some of the protocols described here have also been described in previously published papers (1, 2).

**References**

1. Ishibashi R, Takatsuna Y, Koshizaka M, et al. Safety and efficacy of ranibizumab and luseogliflozin combination therapy in patients with diabetic macular edema: protocol for a multicenter, open-label randomized controlled trial. *Diabetes Ther*. 2020;11:1891–1905.

2. Ishibashi R, Takatsuna Y, Koshizaka M, et al. Ranibizumab with luseogliflozin in type 2 diabetes with diabetic macular oedema: a randomised clinical trial. *Diabetes Obes Metab*. 27:2473–2484.

**Figure 1. Flow diagram of the COMET Trial**

**
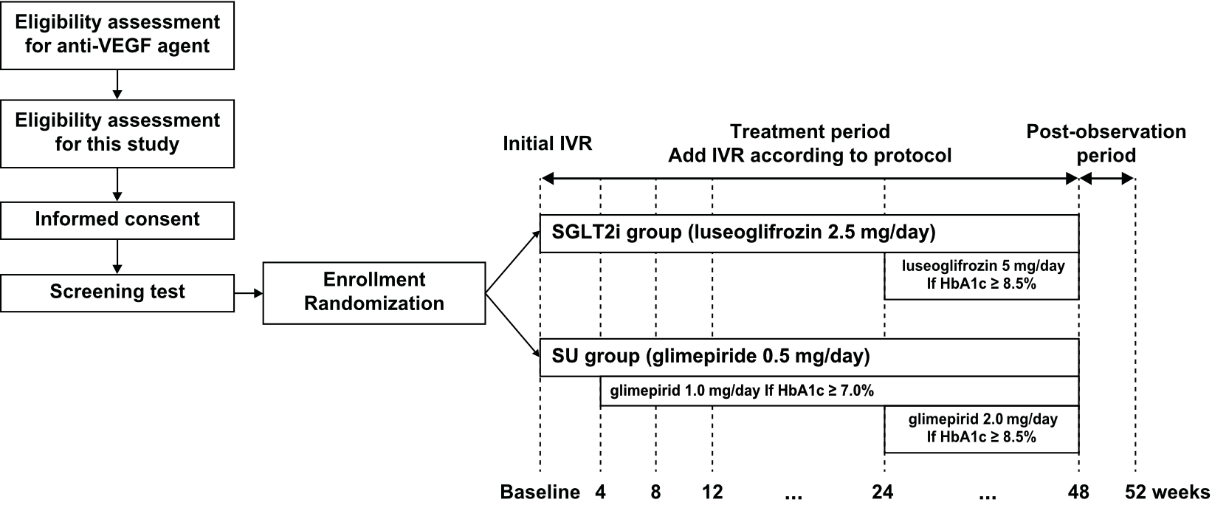
**

Flow diagram of study recruitment, enrolment, randomization, and treatment

IVR, intravitreal injection of ranibizumab; SGLT2i, sodium-glucose cotransporter 2 inhibitor; SU, sulfonylurea; VEGF, vascular endothelial growth factor; HbA1c, glycated hemoglobin

**Table 1. Inclusion and exclusion criteria for the COMET Trial**

| **Inclusion criteria** |
| --- |
| 1. Patients with T2DM aged between 20 and 80 years at the time of providing consent. |
| 2. Patients with DME affecting the fovea, and with BCVA of ≤1.30 (logMAR). |
| 3. Patients with a CRT in the study eye of ≥350 μm measured by OCT at the screening test |
| (if the CRT of both eyes is ≥350 μm, the eye with the thickest retina is defined as the study eye). |
| 4. Patients with HbA1c level ≥6.5% and <12.0% at the screening test. |
| 5. Patients with a BMI ≥18.5 kg/m² at the screening test. |
| 6. Patients with an eGFR ≥30 mL/min/1.73 m² at the screening test. |
| 7. Patients in whom DM treatment has not changed in the 8 weeks preceding the screening test. |
| 8. Patients who provide written consent to participate in this study after a full explanation of the study. |
| **Exclusion criteria** |
| 1. Patients with T1DM. |
| 2. Patients with a history of hypersensitivity to SGLT2 inhibitors or glimepiride. |
| 3. Patients with a history of metabolic acidosis, coma, or precoma due to DM or hypoglycemia within the 24 weeks preceding the screening test. |
| 4. Patients with severe infections, before or after surgery, or severe trauma requiring insulin treatment. |
| 5. Patients with severe renal dysfunction (eGFR<30 mL/min/1.73 m²) or those undergoing dialysis (including peritoneal dialysis). |
| 6. Female patients who are pregnant, possibly pregnant, or planning to be pregnant or breastfeeding. |
| 7. Patients with urinary tract infection or dehydration, or those prone to urinary tract infection or dehydration. |
| 8. Patients with positive urinary ketone bodies (urine qualitative of 2+ or higher). |
| 9. Patients with a history of leg amputation due to leg gangrene. |
| 10. Patients with a history of bone fracture due to osteoporosis. |
| 11. Patients treated with thiazolidinediones, SGLT2 inhibitors, sulfonylureas, or glinides within 8 weeks preceding the screening test. |
| 12. Patients treated with fibrates or diuretic agents who changed the usage or dose within 8 weeks preceding the screening test. |
| 13. Patients who received systemic administration of steroids within 8 weeks preceding the screening test. |
| 14. Patients with a history of cerebrovascular impairment or myocardial infarction within 48 weeks preceding the screening test. |
| 15. Patients with poorly controlled hypertension (blood pressure measured in a sitting position: systolic blood pressure ≥180 mmHg or diastolic blood pressure ≥100 mmHg). |
| 16. Patients with a history of vitrectomy or scleral buckling. |
| 17. Patients with a history of filtration surgery to the study eye for glaucoma treatment or those expected to require filtration surgery  in the future. |
| 18. Patients with active proliferative diabetic retinopathy in the study eye. |
| 19. Patients with a history of idiopathic or autoimmune uveitis in the study eye. |
| 20. Patients with vitreomacular traction syndrome or epiretinal membrane in the study eye (as measured by slit lamp microscope  or OCT) affecting central visual acuity. |
| 21. Patients with iris neovascularization, vitreous hemorrhage, or traction retinal detachment in the study eye. |
| 22. Patients with epiretinal fibrosis in the study eye affecting the macular area. |
| 23. Patients with morphological impairment at the central area of the macula lutea in the study eye, which can affect visual acuity  improvement after the disappearance of macular oedema (including those with atrophy of retinal pigment epithelium, fibrogenesis,  or scarring under the retina, severe retinal ischemia, or structural hard exudates). |
| 24. Patients with a history of cataract surgery or other intraocular surgeries to the study eye within 12 weeks preceding the screening  test. |
| 25. Patients with a history of panretinal laser or macular photocoagulation to the study eye within 12 weeks preceding the screening  test. |
| 1. Patients with a history of yttrium-aluminium-garnet laser posterior capsulotomy to the study eye within 4 weeks preceding the   screening test. |
| 27. Patients who received steroids to the study eye or the study eye surroundings within 16 weeks preceding the screening test. |
| 1. Patients who received angiogenic inhibitors (pegaptanib, bevacizumab, ranibizumab, aflibercept, and others) in any eye   within 12 weeks preceding the screening test. |
| 29. Patients in whom the transparency of optic media in the study eye is insufficient to obtain fundus or OCT images. |
| 30. Patients who received systemic administration of angiogenic inhibitors within 24 weeks preceding the screening test. |
| 31. Patients with complications other than DME that may worsen visual acuity and require surgical intervention during the study period  or affect study endpoints. |
| 32. Patients with signs of infectious blepharitis, keratitis, scleritis, or conjunctivitis in any eye. |
| 33. Patients with a history of hypersensitivity to anti-VEGF agents or fluorescein. |
| 34. Patients who used kallikreinogenase or sairei-to (Chinese medicinal herbal drugs) within 8 weeks before the screening test. |
| 35. Patients with other conditions deemed inappropriate for the study by the investigator/researcher. |

BCVA, best-corrected visual acuity; BMI, body mass index; CRT, central retinal thickness; DM, diabetes mellitus; DME, diabetic macular edema; eGFR, estimated glomerular filtration rate; HbA1c, glycated hemoglobin; OCT, optical coherence tomography; SGLT2, sodium-glucose cotransporter 2; T1DM, type 1 diabetes mellitus; T2DM, type 2 diabetes mellitus; VEGF, vascular endothelial growth factor **Table 2. Observation schedule**

|  | | Obtaining  Consent | Screening  Test | Treatment Period | | | | | | | | | Follow-Up  Period | Discontinuation |  |
| --- | --- | --- | --- | --- | --- | --- | --- | --- | --- | --- | --- | --- | --- | --- | --- |
| （week） | |  |  | 0 [c] | 4 | 8 | 12 | 16 | 20 | 24 | 28,32,36,40,44 | 48 | 52 |  |  |
| Allowable Range (Weeks) | |  | －4～0 | 0 | －１～＋１ | | | －1～＋2 | | | | | －1～＋4 |  |  |
| Ophthalmology | Obtaining Consent | ●* |  |  |  |  |  |  |  |  |  |  |  |  |  |
|  | Participant Background |  | ●  [e] |  |  |  |  |  |  |  |  |  |  |  |  |
|  | Concomitant Medications and Therapies Survey |  | ● | 〇[e] | ● | ● | ● | ● | ● | ● | ● | ● | ● | ● |  |
|  | <Primary Endpoint> | | | | | | | | | | | | | | |
|  | Number of Anti-VEGF Drug Injections |  |  | ● | □[d] | □ | □ | □ | □ | □ | □ | □ |  | □ |  |
|  | <Other Efficacy and Safety Endpoints > | | | | | | | | | | | | | | |
|  | Central Retinal Thickness Measurement |  | ● | 〇 | ● | ● | ● | ● | ● | ● | ● | ● | ● | ● |  |
|  | Subjective and Objective Symptoms |  | ● | ○ | ● | ● | ● | ● | ● | ● | ● | ● | ● | ● |  |
|  | Adverse Events [a] |  |  |  | ● | ● | ● | ● | ● | ● | ● | ● | ● | ● |  |
|  | Vision Test |  | ● | 〇 | ● | ● | ● | ● | ● | ● | ● | ● | ● | ● |  |
|  | Fundus Examination |  | ● | 〇 |  |  |  |  |  | ● |  | ● | ● | ● |  |
|  | Fluorescein Angiography |  | 〇 |  |  |  |  |  |  | 〇 |  | 〇 |  | 〇 |  |
|  | Optical Coherence Tomography Examination |  | ● | 〇 | ● | ● | ● | ● | ● | ● | ● | ● | ● | ● |  |
|  | Intraocular Pressure Test |  | ● | 〇 | ● | ● | ● | ● | ● | ● | ● | ● | ● | ● |  |
| Internal Medicine | Obtaining Consent | ●* |  |  |  |  |  |  |  |  |  |  |  |  |  |
|  | Participant Background |  | ● |  |  |  |  |  |  |  |  |  |  |  |  |
|  | Concomitant Medications and Therapies Survey |  | ● | 〇 | ● |  | ● |  |  | ● |  | ● | ● | ● |  |
|  | Subjective and Objective Symptoms |  | ● | 〇 | ● |  | ● |  |  | ● |  | ● | ● | ● |  |
|  | Adverse Events [a] |  |  |  | ● |  | ● |  |  | ● |  | ● | ● | ● |  |
|  | Height |  | ● |  |  |  |  |  |  |  |  |  |  |  |  |
|  | Weight/Blood Pressure/Pulse |  | ● | 〇 | ● | 〇 | ● | 〇 | 〇 | ● | 〇 | ● | ● | ● |  |
|  | Blood and Urine Tests[b] |  | ● | 〇 | ● |  | ● |  |  | ● |  | ● | ● | ● |  |
|  | Urinary L-FABP |  | ● | 〇 |  |  |  |  |  | ○ |  | ○ |  | ○ |  |
|  | Urinary Alb/Cre Ratio |  | ● | 〇 |  |  |  |  |  | ● |  | ● |  | ● |  |
|  | Electrocardiogram |  | ● |  |  |  |  |  |  |  |  |  |  |  |  |
|  | Insulin |  |  | ● |  |  |  |  |  | ● |  | ● |  | ● |  |
|  | Body Composition/CAVI |  |  | 〇 |  |  |  |  |  |  |  | 〇 |  | 〇 |  |

*Consent was obtained before the screening test. The treatment period commenced within 4 weeks after the screening test.

[a] Adverse events were confirmed from the administration of the study or control drug. The follow-up period tracked adverse events that occurred during the treatment period. [b] Blood tests: WBC, RBC, Hb, Hct, Plt, reticulocyte count (at facilities where possible); Biochemical tests: AST, ALT, γGTP, T-BIL, BUN, Cre, eGFR, TC, HDL-C, LDL-C (indirect method), TG, blood glucose, HbA1c, C-peptide, BNP, UA; and urine tests: general urine tests (urine glucose, urine protein, urine ketone bodies, urinary white blood cells) were measured. [c] For the week 0 evaluation items, the latest data from the screening test to just before the ranibizumab administration was used. [d] □ Conducted under conditions met. [e] ● Mandatory items, ○ Conducted as necessary.

ALT, alanine aminotransferase; AST, aspartate aminotransferase; BNP, brain natriuretic peptide; BUN, Blood urea nitrogen; Cre, creatinine; eGFR, estimated glomerular filtration rate; TG, triglyceride; HbA1c, glycated hemoglobin; Hb, hemoglobin; Hct, Hematocrit; HDL-C, high-density lipoprotein cholesterol; LDL-C, low-density lipoprotein cholesterol; Plt, platelet; RBC, red blood cell; T-Bil, total bilirubin; TC, total cholesterol, UA, uric acid; VEGF, vascular endothelial growth factor; WBC, white blood cell; γGTP, γ-glutamyl transpeptidase;

**Table 3. COMET Trial Investigators**

| **COMET Trial Investigators** |  |
| --- | --- |
| **Chiba University Hospital** | **Ophthalmologist**  Tomoaki Tatsumi, Tomomi Kaiho, Shuichi Yamamoto, Takayuki Baba  **Internist**  Masaya Koshizaka, Koutaro Yokote |
| **Kimitsu Chuo Hospital** | **Ophthalmologist**  Noriko Asaumi, Yosuke Nakamura, Norihiro Shimizu, Tatsuya Nagai, Yuko Hayashi  **Internist**  Ryoichi Ishibashi, Atsushi Takasaki, Hidetoshi　Ochiai, Yusuke Baba |
| **Chiba Rosai Hospital** | **Ophthalmologist**  Yoko Takatsuna, Akihiro Chiba, Katsuya Yagisawa, Azusa Yamagishi  **Internist**  Ko Ishikawa, Masahiro Mimura |
| **Japanese Red Cross Narita Hospital** | **Ophthalmologist**  Yoshihiro Watanabe, Norihiro Shimizu  **Internist**  Kaori Tachibana, Yuki Ohta |
| **National Hospital Organisation Chiba Medical Centre** | **Ophthalmologist**  Miyuki Arai, Kyoko Okada,  **Internist**  Megumi Ohara, Fumio Shimada |
| **Chiba Kaihin Municipal Hospital** | **Ophthalmologist**  Mariko Kubota-Taniai  **Internist**  Kana Watanabe, Aiko Hayashi, Hiyori Kaneko, Takafumi Mayama, Hidetoshi Kawana |
| **Chiba Aoba Municipal Hospital** | **Ophthalmologist**  Akiko Hoshino  **Internist**  Kyohei Yamamoto, |
| **International University of Health and Welfare School of Medicine** | **Ophthalmologist**  Toshiyuki Oshitari  **Internist**  Minoru Takemoto |
| **The Jikei University School of Medicine** | **Biostatisticians**  Sho Takahashi |
| **Keio University Hospital** | **Biostatisticians**  Kengo Nagashima |
